# Differential gene expression analysis after DAPK1 knockout in hepatocellular carcinoma cells

**DOI:** 10.7717/peerj.13711

**Published:** 2022-08-02

**Authors:** Yuanqi Li, Hui Huang, Huajun Yu, Ting Mo, Ting Wei, Guodan Li, Yufang Jia, Xiaoqin Huang, Mingjin Tu, Xiuwen Yan, Haitao Zhang

**Affiliations:** Guangdong Medical University, Zhanjiang, China

**Keywords:** Bioinformatics analysis, DAPK1, Differential gene, Hepatocellular carcinoma, Related gene

## Abstract

**Background:**

The mechanism through which death-associated protein kinase 1 (DAPK1) causes hepatocellular carcinoma (HCC) progression remains unclear. In this study, we aimed to identify key proteins that were altered after DAPK1 knockout.

**Methods:**

Stable DAPK1 knockout HCC cell lines were established, then the differentially expressed genes (DEGs) of HCC were screened using the NetworkAnalyst database and enriched using the Metascape software. Protein-protein interaction networks (PPIs) were analyzed and visualized using the STRING database expansion.

**Results:**

In total, 732 differentially expressed genes were identified, including 415 upregulated genes and 317 downregulated genes. Through Cytoscape software scoring, 10 pivotal genes were found to be closely related to changes in DAPK1 expression; Kininogen-1 (KNG1), Complement C3 (C3), Metalloproteinase inhibitor 1 (TIMP1), and Alpha-2-HS-glycoprotein (AHSG) were the most strongly associated with DAPK1 expression changes. Moreover, western blot analysis results revealed that changes in the levels of proteins encoded by the four key genes after DAPK1 knockout were consistent with those seen in the database screening.

**Conclusions:**

These results provide a direction for further studies on the DAPK1 gene and on the mechanism through which DAPK1 leads to hepatocellular carcinoma development.

## Introduction

Hepatocellular carcinoma (HCC) is one of the most common and fatal malignancies worldwide (Shi et al., 2021), and has high morbidity and mortality rates. Patients with liver cancer are often diagnosed at an advanced stage, thus missing the optimal treatment period, resulting in difficult treatment, poor prognoses and frequent recurrence (Anwanwan et al., 2020). It is therefore important to explore the mechanisms behind the occurrence and development of HCC. DAPK1 is a serine/threonine kinase that acts as an important regulator of apoptosis and inhibits tumor cell growth and metastasis. The dysregulation of DAPK1 expression may lead to cancer development and metastasis (Farag & Roh, 2019). DAPK1 can act as an oncogene or a tumor suppressor gene depending on the cellular environment (Li et al., 2017). Many studies have shown that promoter hypermethylation leads to the loss of DAPK1 expression in numerous tumor types, including chronic lymphocytic leukemia (Bodoor et al., 2014), chronic myeloid leukemia (Qian et al., 2009), diffuse large B-cell lymphoma (Kristensen et al., 2014), and lung cancer (Feng et al., 2008). Cell-based studies have shown that DAPK1 can mediate several types of cell death, including apoptosis (Gade et al., 2012), autophagy (Gozuacik et al., 2008) and necrosis (Wu et al., 2020). The interaction between DAPK1 and cytoskeletal proteins triggers death-related morphological changes in the cell and inhibits cell motility (Ivanovska, Mahadevan & Schneider-Stock, 2014). In addition, DAPK1 can affect the function and survival of stromal cells (Gozuacik et al., 2008).

DAPK1 is a tumor suppressor, and it has been reported that DAPK1 expression levels are low in liver cancer tissues (Katzenellenbogen, Baylin & Herman, 1999). Low levels of DAPK1 mRNA are also associated with a shorter survival period (Li et al., 2017). These findings suggest that the progression of liver cancer is related to the low expression of DAPK1. However, DAPK1 is in liver cancer, especially in common liver cancer cell lines including HepG_2_, PLC/PRF/5, and Hep3B (Zhang et al., 2012). Many studies have reported on some individual mechanisms of DAPK1, but no comprehensive study on the mechanisms of DAPK1 has been done. SB203580 is a mitogen-activated protein kinase (MAPK) inhibitor. Zhang et al. (2012) found that SB203580 induces autophagy in human hepatocellular carcinoma (HCC) cells. However, decreasing DAPK1 can alleviate SB203580-induced autophagy. Understanding specific mechanisms through which DAPK1 affects liver cancer progression could lead to new treatment options. In this study, we analyzed changes in gene expression profiles in liver cancer after DAPK1 was knocked out (Xiang et al., 2020). These results may help explain the specific role of DAPK1 in liver cancer (Gozuacik et al., 2008).

## Methods

### Building DAPK1-knockout Cells

Cas9-DAPK1 knockout plasmids were purchased from Santa Cruz Biotechnology, Inc. PLC/PRF/5 cells were collected and seeded into a six-well plate at a density of 5 × 10^5^ cells/well. The transfection mixture was composed of 2.5 µg Cas9-DAPK1 plasmid and 5 µL Lipo3000 reagent in 125 µL of OpTI-MEM medium. The mixture was added to the wells and mixed evenly. After 24 h, the solution was changed and 2 µg /mL of purinomycin was added. Monoclonal cells were selected using the limited dilution method, and the DAPK1 gene knockout was identified using western blot. The transfection kit was purchased from Thermo Fisher Scientific.

### Western blotting

Cells were seeded in 6-well plates at a density of 5 × 10^5^ cells/well. After the culture medium was discarded, the cells were washed with PBS and 100 µL buffer was added to the wells (protease inhibitor PMSF: RIPA buffer = 1: 99). The cells were scraped and collected into a sterile centrifuge tube. To lyse the cells sufficiently, the cell lysate was pipetted up and down 30 times in a centrifugal tube on ice. After incubating on ice for 30 min, the cell lysate was centrifuged at 15,294 × g for 15 min at 4 °C. The supernatant was collected and the protein concentration was measured. Then, the loading buffer was added to the supernatant and mixed. The mixture was then incubated in boiling water for 10 min. A 10% SDS-PAGE gel was used for electrophoresis and membrane transfer. The transferred membrane was blocked with 5% fat-free milk for 3–6 h (Liu et al., 2020). After rinsing the membrane with TBST (2.4228 g Tris, 8 g NaCl, 1,000 mL H_2_O, 0.6 mL Tween-20) solution, the transferred membrane was incubated with primary antibodies in TTBS solution containing 5% fat-free milk while shaking overnight. The blots were washed in TBST and incubated for 1 h with a 1:2,000 dilution of secondary antibodies. Western blotting was performed using the ECL reagent.

### Immunohistochemistry

The study cohort was consisted of liver samples from 90 hepatocellular carcinoma patients. The specimens were purchased from Shanghai Outdo Biotech Company. This project has been approved by the Review Committee (Ethics Application Ref: YB M-05-02). All participants obtained written consent before surgery. Liver cancer tissues and adjacent tissues were fixed in 10% formalin solution for 24 h, and the moisture in the fixed tissues was removed with graded ethanol. Xylene was used to replace the ethanol in the tissue and make the tissue transparent. The transparent tissue was immersed in melted paraffin wax for soaking and embedding. Under freezing conditions, the tissue was cut into 4 µm thick slices using a microtome. The paraffin sections were dewaxed with xylene. Paraffin sections were soaked with hydrogen peroxide (0.3%) to inhibit endogenous peroxidase activity. After washing with PBS (3.25 g Na_2_HPO_4_.12H_2_O, 0.265 g NaH_2_PO_4_.2H_2_O, 8.766 g NaCl, 1,000 mL H_2_O), these sections were treated with 0.2%–1% Triton-100X at 37 °C for 30 min. The rabbit anti-human DAPK1 (Sigma-D1319, 1:500 dilution) antibody was kept overnight in a humidifying chamber at 4 °C. The specimens were washed with PBS and incubated at 22 ± 3 °C for 1 h with an enzyme-conjugate secondary antibody, and then washed with PBS. All the samples were then stained with both diaminobenzidine and 20% hematoxylin. Images were obtained using a microscope for histopathological analysis.

### Transcriptome analysis

When the cell reached 70–80% confluency, the culture medium was discarded, and the cells were washed with PBS. TRIzol (one mL) was added to each well and incubated for 1 min, after which all TRIzol was transferred to an RNase-free tube. The samples were frozen in liquid nitrogen and stored at −80  °C. After all the samples had been collected, they were transported to Shanghai Applied Protein Technology Co. using dry ice. Samples of each cell were collected separately three times and sent to the sequencing company. Finally, Shanghai Protein Technology Co. extracted RNA from all the cell samples and sequenced them.

### Screening for differential genes

Input data for the gene expression differential analysis were read and the count data was obtained from the gene expression level analysis. DESeq2 (a method for the differential analysis of count data) was used for the differential expression analysis of genes in biological duplicates. The differential analysis was done in triplicate: cell samples were collected three times, RNA was extracted for three times and gene sequencing was performed three times. The *p*-value obtained from the original hypothesis test was corrected during the difference analysis. The revised standard is: *p*-value is less than 0.05, — log2foldchange — greater than 1 as the standard significance of difference. Based on the above analysis results, the volcano map and heat map of differentially expressed genes were drawn, using the NetworkAnalyst database (https://www.networkanalyst.ca/; Xia et al., 2013; Zhou et al., 2019a).

### Pathway annotation and gene ontology enrichment analyses of differential genes

Metascape (https://metascape.org/gp/index.html#/main/step1) integrates more than 40 bioinformatics databases, providing easy access to comprehensive data analysis through a simple interface for a quick one-click analysis (Zhou et al., 2019b). It not only contains a biopathway enrichment analysis, a protein interaction network structure analysis, and a rich gene annotation function, but it also presents the results in a high-quality graphical language that can be easily understood. KEGG (Kyoto Encyclopedia of Genes and Genomes, http://www.kegg.jp/) is one of the databases commonly used for pathway studies (Kanehisa, 2002; Kanehisa et al., 2017). The KEGG database contains information on metabolism, genetic information processing, environmental information processing, cellular processes, biological systems, human disease, and drug development.

### Protein–protein interaction network construction and module analysis

Functional interactions between proteins were analyzed to identify the genes involved with DAPK1. The interactive gene retrieval tool used in this study is Cytoscape software. The protein-protein interaction network (PPI) was constructed using the Cytoscape software by selecting the interactions with a composite score of >0.9 from the search tool to retrieve the interacting genes/proteins (STRING;version 11.5). The Cytoscape software (version 3.7.2) is an open-source bioinformatics database for visualizing molecular interaction networks. The Cytoscape plug-in for molecular complex detection (MCODE) analyzes densely connected regions. The choice criterion for hub genes in this study were as follows: MCODE score of 5, degree cut-off of 2, node score cut-off of 0.2, max depth of 100, and k-score of 2. The KEGG and Gene Ontology (GO) analyses were performed using the Metascape software.

### Selection and analysis of hub genes

The top 10 genes most closely related to DAPK1 were obtained using the MCC algorithm with the Cytoscape plug-in, cytoHubba. The protein expression profile levels (low, medium, and high) of the hub genes of both liver cancer and adjacent tissue were obtained from the Human Protein Atlas (HPA) database. The mRNA expression levels of hub genes in liver cancer and normal subjects were obtained from The Cancer Genome Atlas (TCGA) database.

### MTT assay

PLC/PRF/5 (PLC) cells and DAPK1-knockout PLC/PRF/5 (KO) cells growing in the logarithmic growth phase were digested with trypsin, centrifuged, and suspended in medium. Cells were diluted to 1 × 10^4^cells/mL, and 100 µL of cell suspension was added to 96-well plates. Cells were cultured in CO_2_ at 37 °C. Cells were removed every 24 h and MTT (0.5 g/mL) was added to each well and incubated for 4 h. The cells were then dissolved in DMSO. The light absorption value of each well was measured at 490 nm with a microplate reader to determine cell viability. The above experiment was repeated three times in our laboratory.

### HCC growth in nude mice

A total of 20 mice were randomly divided into two groups (*n* = 10 per group): the PLC group and KO group. PLC or KO cells in the logarithmic growth phase were injected into the PLC group or KO group of mice, respectively. Each mouse was injected with 1 × 10^6^ cells (0.2 mL) under the skin. The mice were raised in barrier facilities with HEPA-filters and fed autoclaved laboratory rodent feed for 21 days. Tumors were resected from the mice in order to weigh and histologically treat. Animal testing in this study strictly followed the guidelines for the Care and Use of Experimental Animals. The mice used in this animal experiment were approved by the Guangdong Animal Experiment Center (approval number: 44007200082541). BALB/ C-NU/NU immunodeficient male mice aged 3–5 weeks were purchased by our laboratory. The average weight of the mice was between 10 and 12 g. The mice were placed in a room reserved for immunosuppressed mice for 2–3 days to help them acclimate to their new environment before each group was inoculated with tumor cells. Twenty-one days post-transplant, 20 mice were euthanized by cervical dislocation and the growing tumors were removed.

### Statistical analyses

The SPSS software (version 20.0; SPSS Inc. Chicago, IL, USA) was used for statistical analysis of the data, and the measurement data were expressed as mean ± standard deviation. In the case of normal distribution and homogeneity of variance, *t*-test was used for data comparison between two groups, and analysis of variance was used for data comparison between multiple groups. For non-normal distribution or uneven variance, Mann–Whitney *U* test was used for comparison of two groups of data, and Kruskal–Wallis H test was used for comparison of multiple groups of data. *P* > 0.05, the difference was not statistically significant; *P* < 0.05, the difference was statistically significant. The statistical graph was made by Graphpad Prism V8.0.2.263 software.

## Results

### Establishment of stable cell lines

**Figure 1:**
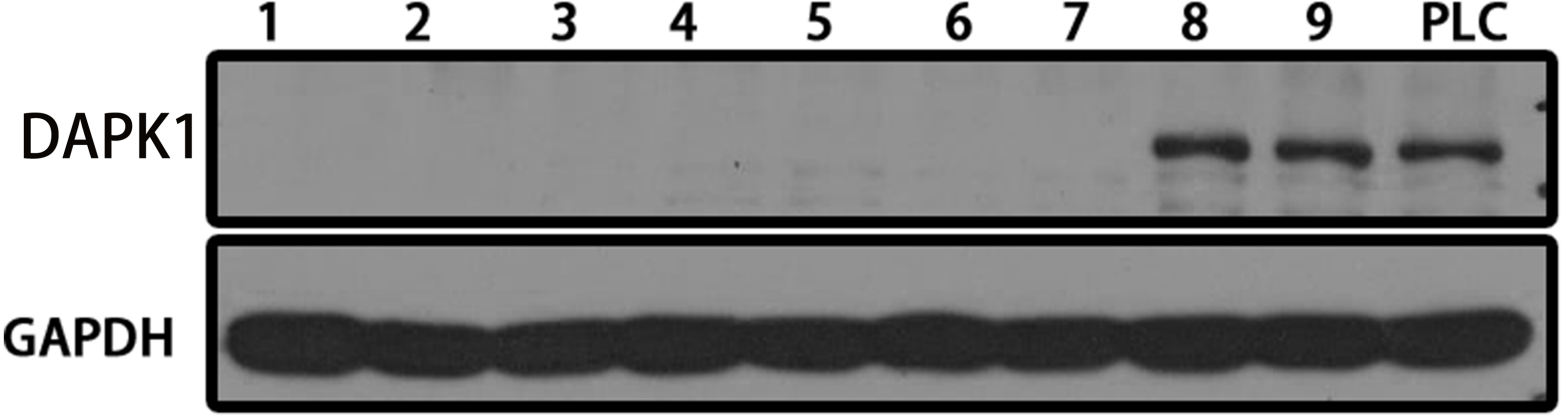
Establishing stable DAPK1-knockout cell lines. After Cas9-DAPK1 plasmid PLC/PRF/5 cell transfection, DAPK1 expression was detected by western blot. PLC, PLC/PRF/5 cells; KO, DAPK1-knockout PLC/PRF/5 cells.

The DAPK1 gene was knocked out from PLC/PRF/5 cells using CRISPR technology. PLC/PRF/5 cells were transfected with the Cas-9-DAPK1 plasmid and screened using a finite dilution method. As shown in Fig. 1A, a total of nine purinomycin-tolerant cell lines were screened, seven of which had the DAPK1 gene knocked out. Those cells that had the DAPK1 knocked out were then cloned and amplified for subsequent experiments.

### Identification of differentially expressed genes (DEGs)

**Figure 2:**
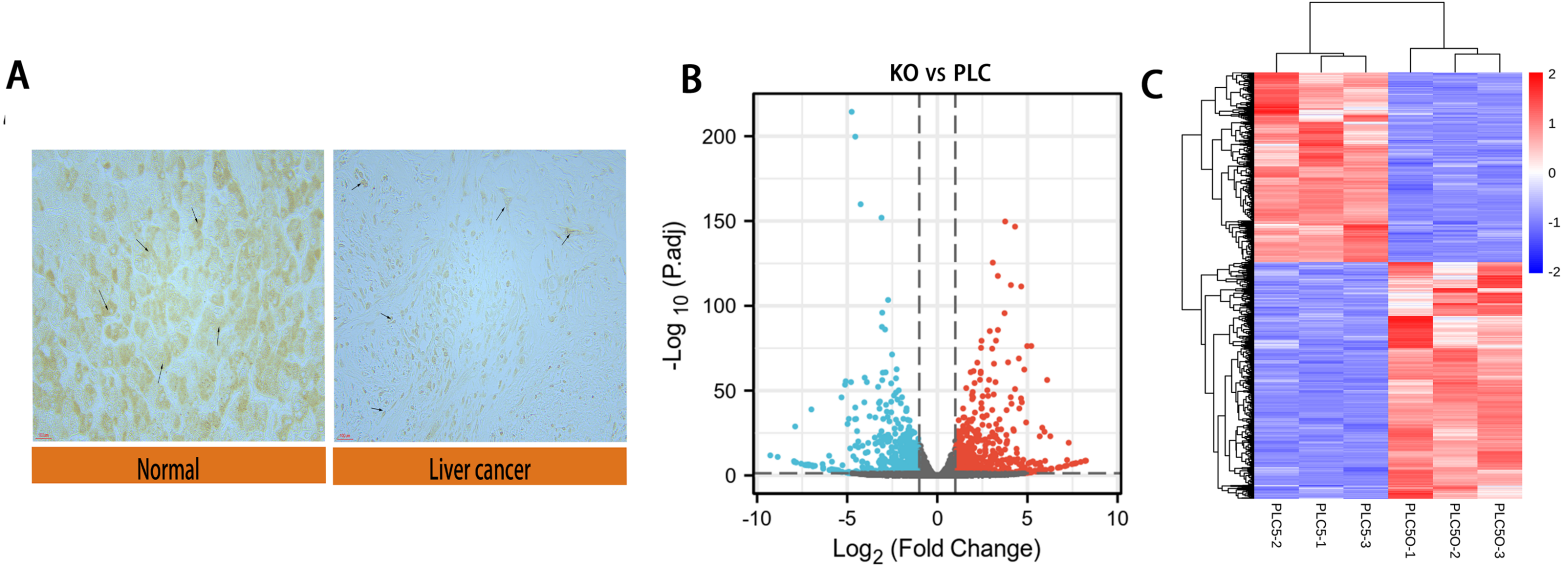
Differential expression of DAPK1 and volcano plot and heat maps of differential genes. (A) Relative expression of DAPK1 compared to normal liver tissue samples. (B, C) Volcano plot and heat map showing the 732 differentially expressed genes. The color red indicates upregulated genes, and blue indicates downregulated genes. PLC, PLC/PRF/5 cells; KO, DAPK1-knockout PLC/PRF/5 cells. padj <0.5, logFC >1.

We performed immunohistochemical experiments on clinically-obtained liver cancer tissues and adjacent tissues; the results are shown in Fig. 2A. The tumor tissue and paracancer tissue of 90 patients with hepatocellular carcinoma were obtained from clinical practice, in which 70 groups had obvious expression difference and 20 groups had no obvious expression difference. The results showed that the expression of DAPK1 in adjacent tissues was lower than that in the liver cancer tissues. For samples that were biological replicates, we used DESeq2 in the NetworkAnalyst database for differential gene expression analysis. A total of 732 DEGs were identified, including 415 upregulated genes and 317 downregulated genes, as shown in the volcano map and heat map (Figs. 2B–2C).

### KEGG and Gene Ontology enrichment analyses of DEGs

**Figure 3:**
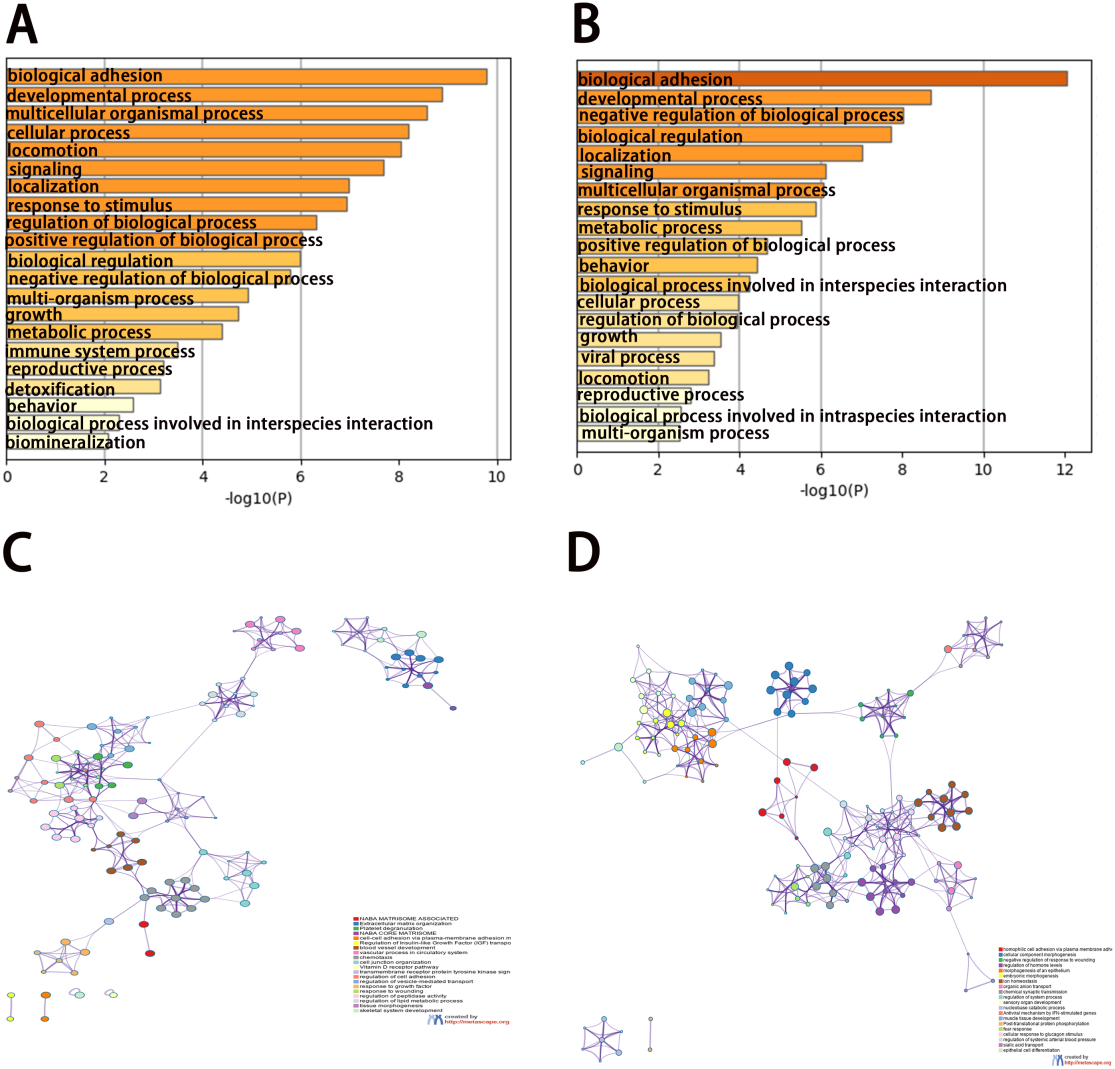
Enrichment analysis of differentially expressed genes (DEGs). (A, B) Functional enrichment analysis of DEGs. Bar graph showing the top 20 results from enrichment analyses of upregulated and downregulated genes.. *P* value is shown in color. (C, D) The network of enriched terms of upregulated and downregulated genes, showing the top 20. Each cluster ID is indicated with a specific color.

To analyze the features of the DEGs, we performed a GO enrichment analysis of upregulated and downregulated genes using the Metascape database. The results showed that the upregulated genes were mainly enriched in naba matrisome associated, extracellular matrix organization, platelet degranulation, naba core matrisome, and cell–cell adhesion via plasma-membrane adhesion molecules (Figs. 3A and 3C). The downregulated genes were significantly enriched in homophilic cell adhesion via plasma membrane adhesion molecules, cellular component morphogenesis, negative regulation of response to wounding, regulation of hormone levels, and morphogenesis of the epithelium (Figs. 3B and 3D). The results of the KEGG pathway analysis demonstrated that both the upregulated genes and downregulated genes were mainly enriched in biological adhesion.

### PPI network construction

**Figure 4:**
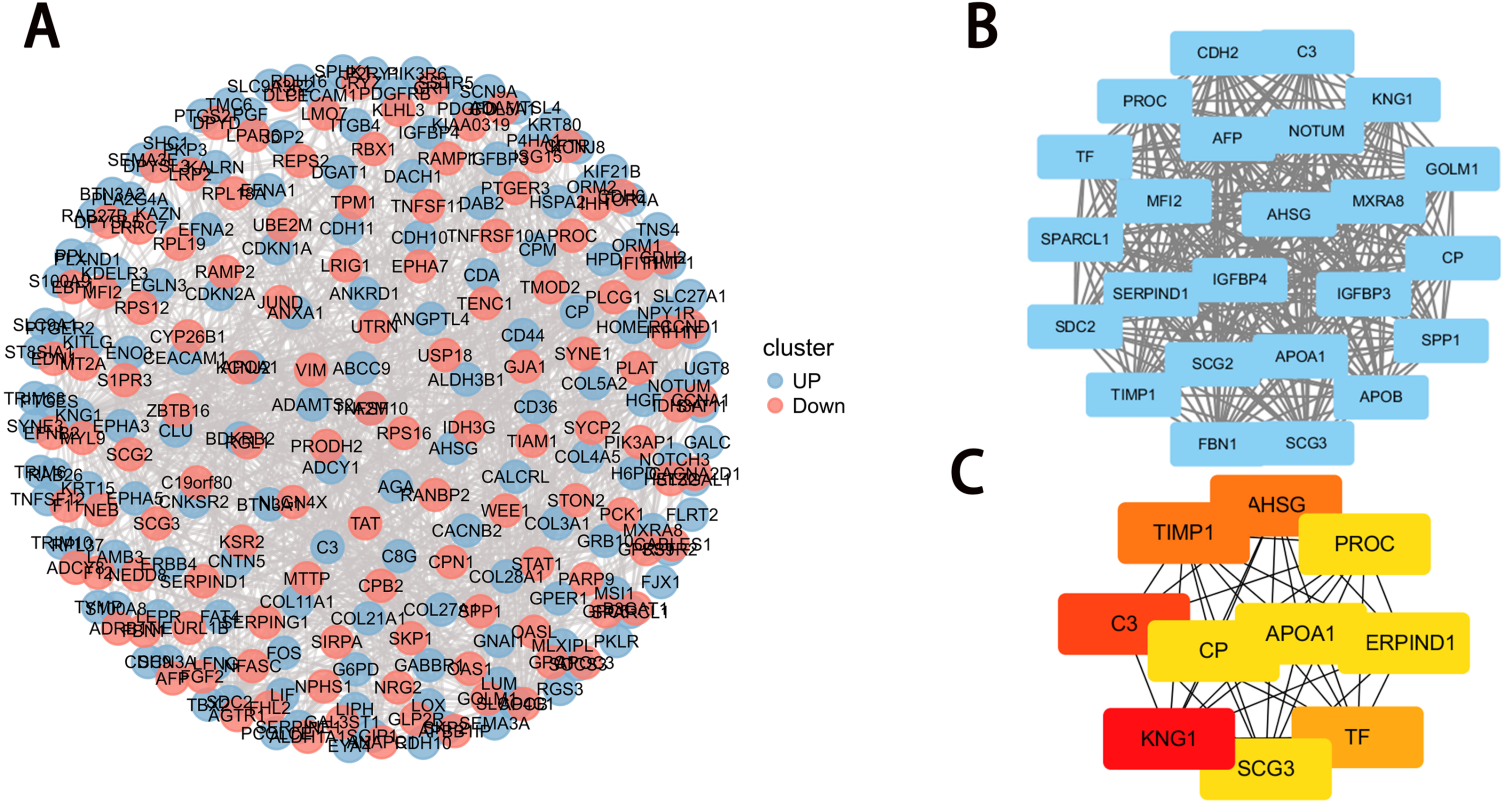
PPI network construction and module analysis. (A) PPI network of DEGs. The upregulated genes are marked in red, while the downregulated genes are marked in blue; (B) the densest connected regions (24 nodes, 276 edges) in the PPI network were identified using Cytoscape. (C) Ten hub genes were identified in the densest connected regions with the MCC algorithm, using cytoHubba. The score is indicated in the color red. A darker color indicates a higher score.

The PPI network of DEGs was first obtained from the STRING database and then the densest connection region (24 nodes, 276 edges) was analyzed using the Cytoscape database (Figs. 4A–4C). Functional enrichment analysis of genes in this dense region showed that these genes were mainly enriched in post-translational protein phosphorylation, hemostasis, complement and coagulation cascades, positive regulation of NABA ECM glycoprotein, and lipid localization (Table 1).

**Table 1:** Ten hub genes and their functions.

**Gene symbol**	**Description**	**Protein function (Protein Atlas)**
KNG1	kininogen 1	Predicted secreted proteins; Disease related genes
C3	complement C3	Candidate cardiovascular disease genes; FDA approved drug targets: Biotech drugs; Predicted intracellular proteins; Predicted secreted proteins; Disease related genes
TIMP1	TIMP metallopeptidase inhibitor 1	Cancer-related genes: Candidate cancer biomarkers; Candidate cardiovascular disease genes; Predicted secreted proteins; Predicted intracellular proteins
AHSG	alpha 2-HS glycoprotein	Cancer-related genes: Candidate cancer biomarkers; Predicted secreted proteins
TF	transferrin	Cancer-related genes: Candidate cancer biomarkers; Predicted intracellular proteins; Predicted secreted proteins; Disease related genes
APOA1	apolipoprotein A1	Cancer-related genes: Candidate cancer biomarkers; Candidate cardiovascular disease genes; Predicted intracellular proteins; Predicted secreted proteins; Disease related genes
SERPIND1	serpin family D member 1	Candidate cardiovascular disease genes; FDA approved drug targets: Biotech drugs; Predicted secreted proteins; FDA approved drug targets: Small molecule drugs; Disease related genes
SCG3	secretogranin III	Predicted intracellular proteins; Predicted secreted proteins
PROC	protein C, inactivator of coagulation factors Va and VIIIa	Cancer-related genes: Candidate cancer biomarkers; Candidate cardiovascular disease genes; FDA approved drug targets: Biotech drugs; Predicted intracellular proteins; Predicted secreted proteins; FDA approved drug targets: Small molecule drugs; ENZYME proteins: Hydrolases; Enzymes; Disease related genes; Peptidases:Serine-type peptidases
CP	ceruloplasmin	Cancer-related genes: Candidate cancer biomarkers; Candidate cardiovascular disease genes; Predicted intracellular proteins; Predicted secreted proteins; Enzymes; ENZYME proteins:Oxidoreductases; Disease related genes; Potential drug targets

### Selection of hub genes

The MCC algorithm was used to obtain the top 10 genes from the PPI network using the Cytoscape plug-in cytoHubba. The ten key genes were: KNG1, TIMP1, Alpha 2-HS glycoprotein AHSG, TF, SCG3, CP, PROC, APOA1, C3, and SERRIND1, of which KNG1, TIMP1, AHSG and C3 had the highest correlation score with DAPK1 indicating that they were closely related to DAPK1. This study focused on the relationship between these four key genes and the DAPK1 gene.

### Analysis of four hub genes

**Figure 5:**
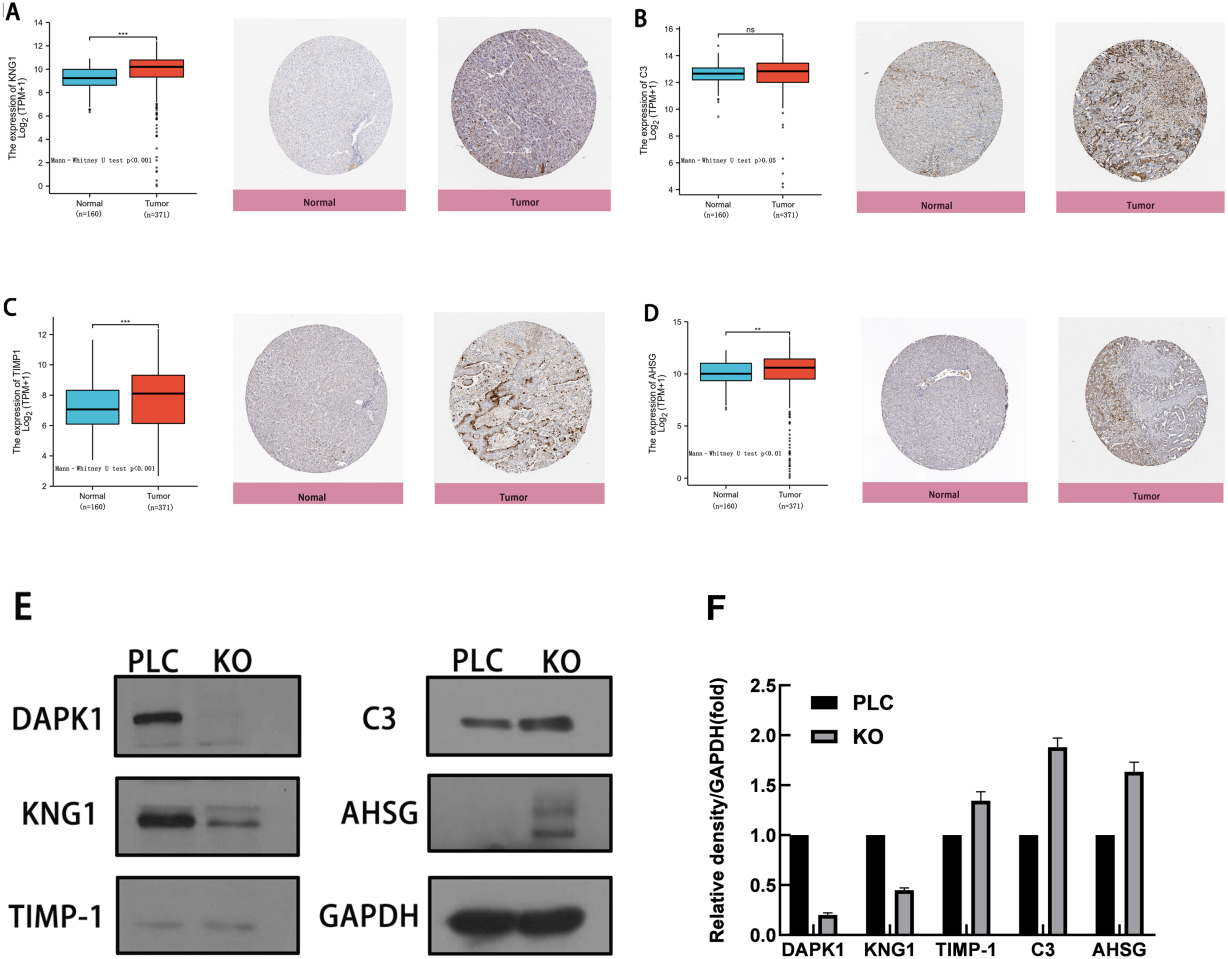
Comparison of gene expression and protein expression of four hub genes. (A, B, C, D) Relative expression of KNG1, C3, TIMP1, and AHSG in HCC, compared to normal liver tissue samples. ns, *p* ≥ 0.05; *, *p* < 0.05; **, *p* < 0.01; ***, *p* < 0.001; ****, *P* < 0.0001. On the left is the mRNA expression levels of KNG1, C3, TIMP1, and AHSG, and on the right is the protein expression levels. (E) Validation of four genes in PLC/PRF/5 cells and DAPK1-knockout PLC/PRF/5 cells. PLC, PLC/PRF/5 cells; KO, DAPK1-knockout PLC/PRF/5 cells.

To evaluate the expression of these four genes in tissues, images of human HCC tissues were obtained from The Human Protein Atlas and compared with the mRNA expression data of the four genes provided by The Cancer Genome Atlas. As shown in Figs. 5A–5D, the results of the analysis of data from these two databases were consistent. Compared with normal tissues, KNG1, TIMP1, and AHSG expression was elevated. C3 mRNA and protein expression levels were also increased, but these results were not statistically significant.

The proteins were extracted from PLC/PRF/5 cells and KO cells for WB verification, as shown in Figs. 5E. 5F shows the normalized results of the western blot analysis and the statistical analysis using mean and standard deviation methods, shown with a bar chart. The levels of C3, TIMP1, and AHSG increased in DAPK1-knockout cells, whereas the level of KNG1 decreased.

### Inhibition of PLC/PRF/5 growth by DAPK1

**Figure 6:**
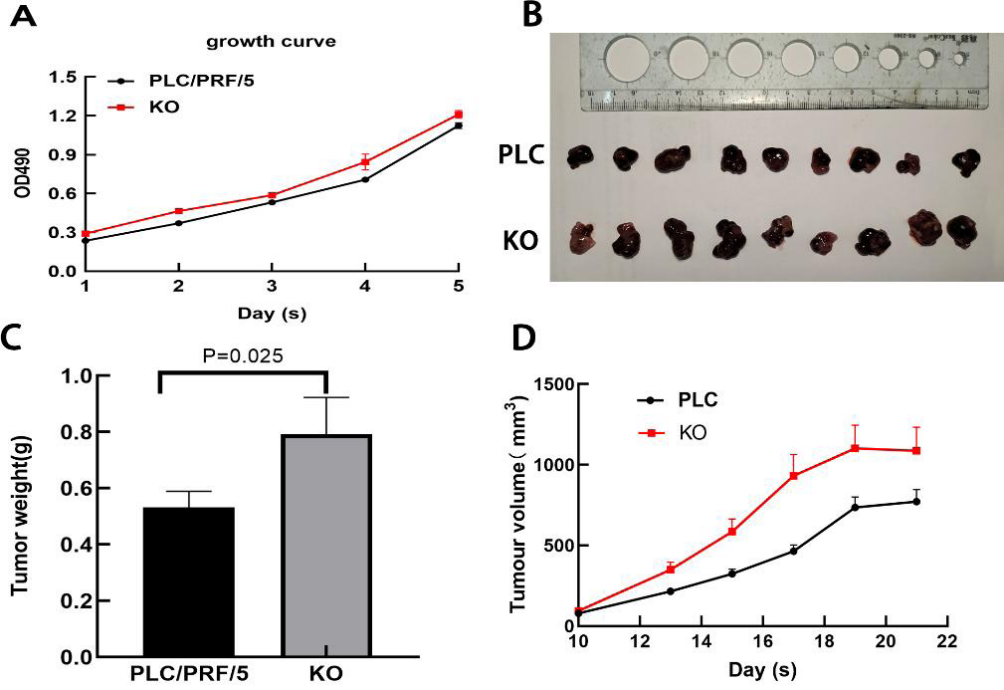
Results of animal experiments. (A) Growth curves of two stable cell lines. (B) The image of dissected tumors from nude mice. (C) Tumor weight histogram from nude mice. (D) Tumor volume growth curves in nude mice. PLC, PLC/PRF/5 cells; KO, DAPK1-knockout PLC/PRF/5 cells.

Next, the effect of DAPK1 expression on PLC/PRF/5 cell growth *in vitro* was assessed. MTT assay results showed that KO cells proliferated faster than PLC cells (Fig. 6A). Furthermore, we performed *in vivo* tumorigenesis experiments to confirm that DAPK1 inhibits PLC/PRF/5 cell proliferation. The results showed that the tumor volume and size in mice inoculated with PLC/PRF/5 cells were significantly less than in those inoculated with KO cells (Fig. 6B). The tumor growth rate and tumor weight of mice inoculated with PLC/PRF/5 cells were significantly higher than in those inoculated with KO cells (Fig. 6C and Fig. 6D).

## Discussion

Clustered regularly spaced short palindromic repeats (CRISPR), short hairpin RNA (shRNA), and CRISPR interference (CRISPRi) are knockout screening technologies for essential genes. Bastiaan Evers demonstrated that CRISPR technology outperforms shRNA and CRISPRi screening technologies (Evers et al., 2016). The CRISPR technology performed better, with lower noise than the other two technologies. More importantly, its off-target effects were minimal. CRISPR is a natural defense system unique to bacteria and archaea that helps identify the genetic material of infectious organisms (Zhang, 2021). This technique has been widely used worldwide (Zhang et al., 2021). In this study, the CRISPR/Cas9 system was used to target DAPK1 gene knockout in PLC/PRF/5 cells to study the specific mechanism underlying HCC progression. DAPK1 knockout was observed in seven of the nine colonies screened. These seven colonies were amplified and used for subsequent experiments (Fig. 1).

Gene expression and protein interactions were analyzed to identify potential key genes associated with DAPK1. An analysis of the sequencing data of PLC/PRF/5 cells and DAPK1-Knockout PLC/PRF/5 cells revealed 732 differentially expressed genes in DAPK1-knockout cells compared with PLC/PRF/5 cells (Fig. 2). We then constructed a PPI network to detect the internal relationship of differentially expressed genes (Figs. 3 and 4), and identified the following 10 hub genes using Metascape: KNG1, C3, TIMP1, AHSG, TF, APOA1, SERPIND1, SCG3, PROC, and CP (Fig. 4). According to the Cytoscape plug-in, cytoHubba, KNG1, C3, TIPM1, and AHSG were found to have the strongest correlation with DAPK1 (Fig. 4). We found that all four key genes have a similar protein function, which is predicted to be secreted proteins (Table 1).

Complement C3 (C3) is a complement protein that plays an important role in natural immunity and is produced by activated macrophages in the liver, fat cells, as well as inflammatory sites (Onat et al., 2011). Zarkadis, Mastellos & Lambris (2001) have shown that elevated C3 levels are a marker of cardiometabolic disorders. Serum complement C3 levels in HCC patients are higher than those in healthy subjects and patients with hepatitis B virus (HBV) infection (Ali, Abo-Shadi & Hammad, 2005). These studies showed that the expression of C3 increases after the malignant transformation of liver cells. We noted an increase in C3 expression when DAPK1 was knocked out in PLC/PRF/5 cells. HCC deterioration increased after DAPK1 knockout, which may be the reason for the higher expression of C3 in the DAPK1-knockout PLC/PRF/5 cells than in the PLC/PRF/5 cells (Fig. 5) (Zhu et al., 2018).

Alpha 2-HS glycoprotein (AHSG), a serum glycoprotein produced predominantly by hepatocytes, is an important biomarker of near-term mortality in patients with cirrhosis and HCC (Wang et al., 2009). Previous studies have demonstrated that AHSG is highly expressed in liver cancer tissues compared to adjacent non-tumor tissues (Xiang et al., 2015), which is consistent with our screening results. Xiang et al. (2015) demonstrated that AHSG knockout can inhibit proliferation in HepG_2_ and BEL7402 cell lines, suggesting that the aberrant expression of AHSG may be related to the occurrence of HCC. Xiang et al. (2015) also used an itRAQ-based secretory analysis to show that the upregulation of AHSG in drug-resistant MDR cell lines may be a predictor of chemotherapy resistance in HCC. DAPK1 is an independent prognostic indicator of liver cancer, and the progression of liver cancer increases when DAPK1 is poorly expressed. In this study, when DAPK1 was knocked out, AHSG expression increased (Fig. 5) (Huang et al., 2018; Swallow et al., 2004).

Kininogen-1 (KNG1), a cysteine proteinase inhibitor, is encoded by the KNG1 gene, which is abnormally expressed in HCC tissues and is a potential marker for HCC (Jiang et al., 2019). The low expression of KNG1 in cancer patients increases the vitality of cancer cells and plays a crucial role in carcinogenesis (Kawasaki et al., 2003; Xu et al., 2018). The degree of plasma kinkinin reduction is directly related to the severity of liver function impairment, and KNG1 levels are lower in patients with hepatic fibrosis/cirrhosis than in those with non-hepatic fibrosis C (Henkel et al., 2011). The degree of liver function impairment in patients with liver cancer is generally severe, and the KNG1 level is also low. After DAPK1 knockout, cell proliferation increased, liver function was further impaired, and KNG1 levels decreased (Fig. 5). Moreover, KNG1 had the highest association with DAPK1 in the PPI network, suggesting that the role of DAPK1 may be further correlated with KNG1.

Tissue inhibitor of metalloproteinase 1 (TIMP-1) is a protein that is structurally related to the zinc-dependent enzyme family. The overexpression of TIMP-1 has been confirmed to increase the migration of liver cancer cells (Roeb et al., 2005), and inhibit apoptosis in tumor cells (Li, Fridman & Kim, 1999). Previous studies have demonstrated that liver cancer patients with lower serum TIMP-1 concentrations have significantly better overall survival than those with higher serum TIMP-1 concentrations (Roeb et al., 2005). After DAPK1 was knocked out, TIMP-1 expression increased (Fig. 5) in PLC/PRF/5 cells and PLC/PRF/5 cell proliferation also increased (Fig. 6).

*In vivo* tumorigenesis confirmed that DAPK1 knockout significantly promoted tumor cell growth (Fig. 6). Previous studies have shown that a high expression of C3, TIMP-1, and AHSG genes in tumors can promote tumor cell proliferation and tumor deterioration (Chen et al., 2014). A low expression of KNG1 in tumors inhibits the occurrence and development of cancer and inhibits apoptosis. These four genes were correlated with changes in expression after DAPK1 knockout. In conclusion, suppression of DAPK1 may accelerate tumor growth and result in the upregulation of C3, TIMP-1, and AHSG expression and downregulation of KNG1 expression.

## Supplemental Information

10.7717/peerj.13711/supp-1 Supplemental Information 1 Raw data

10.7717/peerj.13711/supp-2 Supplemental Information 2 The experimental process

10.7717/peerj.13711/supp-3 Supplemental Information 3 Author Checklist - Full

## Additional Information and Declarations

### DNA Deposition

The following information was supplied regarding the deposition of DNA sequences: The sequences are available at GenBank: PRJNA798901.

### Data Availability

The following information was supplied regarding data availability: The raw data is available in the Supplementary Files.
