# Supplementary material for: Differential gene expression analysis after DAPK1 knockout in hepatocellular carcinoma cells
Source: PeerJ. 2022 Aug 2;10:e13711. doi: 10.7717/peerj.13711 (PMC9354754; doi:10.7717/peerj.13711)
Supplement: Supplemental Information 2 [file peerj-10-13711-s002.docx]

**The experimental process**

1. In the first step, the DAPK1 gene was knocked out by CRISPR technology to construct a stable cell line.
2. The second step was to send the constructed stable cell lines to the company for sequencing to find the altered genes after DAPK1 was knocked out.
3. The database was used to draw biogenic heat maps and volcanic maps to identify the differential genes.
4. Enrichment analysis, classification of differential genes.
5. MCC algorithm was used to obtain the first 10 genes through Cytoscape plug-in cytoHubba, and select the most critical genes related to DAPK1.
6. WB test to verify the relationship between these genes and DAPK1 as the database screening.
7. Animal experiments, MTT experiments, clone plate experiments, and immunohistochemistry experiments all confirmed that the progression of liver cancer was increased after DAPK1 knockdown
